# Advancement in the molecular perspective of plant-endophytic interaction to mitigate drought stress in plants

**DOI:** 10.3389/fmicb.2022.981355

**Published:** 2022-09-02

**Authors:** Prafull Salvi, Himanshu Mahawar, Ruchi Agarrwal, Kajal, Vibhav Gautam, Rupesh Deshmukh

**Affiliations:** ^1^Agriculture Biotechnology Department, National Agri-Food Biotechnology Institute, Mohali, India; ^2^ICAR-Directorate of Weed Research, Jabalpur, MP, India; ^3^National Research Center on Pomegranate, Solapur, India; ^4^Centre of Experimental Medicine and Surgery, Institute of Medical Sciences, Banaras Hindu University, Varanasi, India; ^5^Plaksha University, Mohali, India

**Keywords:** endophyte, drought stress, crop improvement, abiotic stress, omics, plant-microbe interaction

## Abstract

Change in global climate has started to show its effect in the form of extremes of temperatures and water scarcity which is bound to impact adversely the global food security in near future. In the current review we discuss the impact of drought on plants and highlight the ability of endophytes, microbes that inhabit the plants asymptomatically, to confer stress tolerance to their host. For this we first describe the symbiotic association between plant and the endophytes and then focus on the molecular and physiological strategies/mechanisms adopted by these endophytes to confer stress tolerance. These include root alteration, osmotic adjustment, ROS scavenging, detoxification, production of phytohormones, and promoting plant growth under adverse conditions. The review further elaborates on how omics-based techniques have advanced our understanding of molecular basis of endophyte mediated drought tolerance of host plant. Detailed analysis of whole genome sequences of endophytes followed by comparative genomics facilitates in identification of genes involved in endophyte-host interaction while functional genomics further unveils the microbial targets that can be exploited for enhancing the stress tolerance of the host. Thus, an amalgamation of endophytes with other sustainable agricultural practices seems to be an appeasing approach to produce climate-resilient crops.

## Introduction

In the light of current scenario of climate change, the net damage cost to the ecosystem is incessantly increasing. The exponential rise in global population, and the subsequent anthropogenic activities further augment the magnitude of these catastrophic situations. These situations severely affect the ecological system, including plants, animals, and microbes (Ahmed, 2020; Morris et al., 2020; Lal, 2021). However, due to their mobile nature, animals are capable of avoiding the exposure to these inimical situations. On the contrary, the sedentary nature of plants inevitably encounters them with calamitous impact of the climate change such as reduced precipitation, extreme temperature, and light (Kaur et al., 2021; Manna et al., 2021). Such encounter affects the plant life cycle at their different developmental stages and eventually results in loss of crop productivity. As the global livelihood/economy is copiously dependent on the agricultural output, an urgent action is required to tackle the situation.

Consequently, to deal with this conundrum plants have evolved different strategies that alter their physiological, biochemical and molecular facet during stress exposure to sustain their growth (Bechtold and Field, 2018). Molecular responses are majorly driven by growth regulators that dramatically alter the transcriptional regulation and cell signaling. The action of phytohormonal and transcriptional syndicate is orchestrated by the dynamic signaling cascade which in turn exhibit a feedback regulation (Banerjee and Roychoudhury, 2018; Xie et al., 2019; Li et al., 2020; Salvi et al., 2021; Gandass et al., 2022). An adequate signaling event is a prerequisite for the appropriate regulatory response against the stressor to adapt to the adversities. In plants, the osmotic adjustment and reactive oxygen species (ROS) homeostasis are the key cellular responses to minimize cellular damage (Suzuki and Katano, 2018; Nadarajah, 2020; Takahashi et al., 2020). These responses are largely reliant on different factors such as plant age and developmental stage; stress type, severity, and duration (Kazemi-Shahandashti and Maali-Amiri, 2018; Berens et al., 2019; Kumar et al., 2019). Several efforts to improve stress response in crop plants have been carried out using genetic engineering and molecular breeding approaches. However, to attain a sustainable crop improvement, the intervention of endophytic microbes to mediate improved stress response has emerged as an appeasing approach.

Endophytes are non-pathogenic microbes residing inside the plant tissue asymptomatically. Endophytic microbiome elicits different local and systemic responses in plants that often facilitate host growth by modulating metabolic events for mutual benefits. The modulation of the metabolic events may result in the accumulation of osmolyte, ROS scavenging, phytohormone production, phosphate solubilization, enhanced nutrient availability, pathogen suppression, and many more (Brader et al., 2014; Arora and Ramawat, 2017; Trivedi et al., 2020; Mattoo and Nonzom, 2021; Orozco-Mosqueda et al., 2021). Moreover, they also facilitate the production/accumulation of several bioactive compounds that contribute toward the (a) biotic stress tolerance response in the host plant (Lata et al., 2018; Mengistu, 2020; Morelli et al., 2020; Suryanarayanan and Shaanker, 2021). The endophyte-associated stress mitigation is largely dependent on the host environmental niche, for instance, the microbiota of plants from hot springs and coastal areas appear to endow heat and salinity tolerance, respectively (Rodriguez et al., 2008; Nanjundappa et al., 2021). This could be due to their ability to produce osmo-protective molecules like proline, melatonin, and carotenoid during abiotic stress exposure (Pacifico et al., 2019). Interestingly, such stress response is quite specific, as the bacteria from one locale appears to be incapable of imparting tolerance to other. Besides, owing to their metabolic drive to produce defense molecules such as proteases, siderophore, and chitinase, several endophytes exhibit antagonistic activity against the phytopathogen growth. In light of recent research, the use of endophytes as a biocontrol agent against phytopathogen and herbivores or to confront the environmental stressor such as oxidative, drought, and salinity stress has emerged as a promising strategy. Conventionally, the plant-microbe interaction is ubiquitous, and it may have a beneficial or hostile impact on the host plant. However, studies have been conducted to understand and deal with the negative impact of microbes, that has been already reviewed and documented earlier (Rai and Agarkar, 2014; Brader et al., 2017).

Apart from endophytic bacteria and fungi, asymptomatic viruses also have important role in alleviating the abiotic stress in plants. Tomato yellow leaf curl virus (TYLCV), belonging to Begomovirus family, has been found to impart tolerance to tomato plant against severe drought stress (Gorovits et al., 2019; Shteinberg et al., 2021). Upon infection, the major TYLCV proteins interact with the heat shock transcription factor HSFA2 and suppress the heat shock response. This is facilitated by inhibition of HSFA2 translocation to nuclei, which further prevents downregulation of heat responsive genes. TYLCV inhibits the HSP90 (HSFA1 and HSFB1) and SGT1 (co-chaperone) functions in tomato plants, thus suppressing host cell death. Additionally, the TYLCV also aids by redirecting some principal amino acids and carbohydrates from above ground parts to roots, thus mitigating effects of drought.

Although in response to (a) biotic stresses, plant cell exhibits a dynamic yet highly regulated response to mitigate the stressful conditions, sometimes the inhabitant symbiont endophytes in the host appears to have either direct or indirect influence on the stress signaling. Such endophyte-mediated signaling cascade apparently affects the expression of stress-responsive genes by implicating the phytohormonal syndicate or transcriptional module. However, it is important to comprehend the molecular basis of plant-endophyte relation and the mechanism underlying the stress response mediated by endophytes. The main aim of this review was to provide a comprehensive overview of the impact of drought on plant physiology and how endophytes (bacteria and fungi) can play a pivotal role in mitigating this stress in their hosts. For this the reader is first given an idea of the endophytism and then the mechanisms employed by endophytes to confer stress tolerance to host are discussed. Further, we highlight the recent studies where omics-based approaches have broadened our understanding of endophyte-mediated drought tolerance in host.

## Endophytism: An overview

The term “endophyte” was first introduced by De Bary in 1866 which implies “inside the plant.” Endophytes can potentially be prokaryotic or eukaryotic microbes such as archaea, bacteria, fungi or viruses which dwell inside the host asymptomatically (Bacon et al., 2000). They reside in different parts of the host plants throughout their entire life cycle or during some part of it. They can either transfer from generation to generation through vertical or horizontal transmission or arise from the rhizosphere or phyllosphere. Plants release molecules such as flavonoids, lipo-chitooligosaccharides, strigolactone (Rozpadek et al., 2018), arabinogalactan (Nguema-Ona et al., 2013), and many more which act as a signal for endophytic colonization. To penetrate inside the host plant, endophytes cross the first line of defense of the plant immune system by recognizing the conserved molecules termed as microbe-associated molecular patterns (MAMPs). Some of the elicitors which work as MAMPs are peptidoglycans, lipopolysaccharides, flagellin, chitin, bacterial SOD, beta-glycan bacterial cold shock proteins, and β-glucan from oomycetes (Newman et al., 2013). Specific receptors termed as pathogen recognition receptors (PRRs), present on the surface of plant cells, recognize MAMPs. Some endophytes synthesize and release hydrolytic enzymes such as pectinase, xylanase, cellulase, and proteinase and penetrate inside the host plant.

Being in mutualistic interaction with the host plant, endophytes aids in their nutrient uptake, tolerance to abiotic and biotic stresses, regulation of hormone pathways, etc. In turn, the endophyte also receives favors from the host plant. The host allows endophytes to colonize the suitable niches to multiply bypassing host’s autoimmune system, besides providing carbon for energy and other nutrients. However, the colonization, distribution and endophyte diversity are regulated by host genotype and ecology. In number of studies undertaken to elucidate plant-endophyte interaction, it was concluded that diverse morphology, physiology, habitat and metabolites of host plants govern their potential to employee various endophytes (Wu et al., 2021). In other words, endophytes smartly monitor their structure and diversity in lieu of various host genotype, plant parts, growth stages, etc., to maneuver regular nutrient acquisition for their own growth and propagation.

Upon colonization of host plant tissues, endophytes get exposed to the micro-environment inside the host. Since plants are constantly exposed to differences in external environment, the endophytes inhabiting them should also be highly adaptive. Consequently, endophytic communities exhibit high variability and are dynamic during plant development (Borruso et al., 2018). The ability of the endophytic species to adapt and their interaction with other microbial community plays a decisive role in successful establishment of endophytes within the host plants. Molecular or cellular changes in endophytic microorganisms is a reflection of its response to external stimuli in the plant. For example, a change in redox state of plants as a response to osmotic stress results in a corresponding change in endophyte gene expression patterns (Sheibani-Tezerji et al., 2015) community. Similarly, hydrogen peroxide breakdown by seed colonizing bacteria is probably an adaptation strategy of the bacteria to the changing redox conditions during germination (Gerna et al., 2020). Therefore, for successful colonization by endophytes they need to possess several adaptive strategies and should be able to ace the changing micro-environment inside the plant tissue to be able to live in a mutualistic symbiotic association with their host plant.

Studies show that the genome of endophytes consist of information that codes for traits favorable to their host plants (Orozco-Mosqueda del Carmen et al., 2021). The mutualistic association of plant and endophytes result in the production of several bioactive compounds (Gouda et al., 2016; Keshri et al., 2021; Verma et al., 2022) such as artemisin (Li et al., 2012), camptothecin (Zhang et al., 2012), helvolic acid (Prasad et al., 2014), taxol (Heinig et al., 2013), huperzine (Nair and Padmavathy, 2014), and azadirachtin (Kusari et al., 2012) which are beneficial for medicine, agriculture, biodegradation, and bioremediation sectors. In response to biotic stress, endophytes produce several anti-bacterial, anti-fungal proteins to protect plants from phytopathogens. Endophytes play an important role in the growth of their host plants by increasing the availability of several nutrients such as phosphorus, potassium, and zinc, by fixing atmospheric nitrogen, and by synthesizing phytohormones, siderophores, hydrogen cyanide, ammonia, proline, carotenoids, melatonin, etc., Many transcriptomic and metabolomic studies have indicated that several plant growth-promoting pathways in plants are associated with endophytic gene products. Endophytes mitigate metal phytotoxicity through biotransformation, extracellular precipitation, intracellular accumulation, and transformation of toxic metal ions into non-toxic or less toxic forms (Ojuederie and Babalola, 2017). To conclude, endophytes are involved in phytoremediation, defense against phytopathogens, production of bioactive compounds, and promote plant growth. These activities of endophytes make them better biocontrol agent and bio-inoculant that can be best used as an alternative to chemicals (pesticides/fertilizers) to attain sustainable agriculture practice.

## Implication of endophytic microbes in mediating the dehydration stress response in host plants

In order to comprehend the role and regulatory aspect of stress response facilitated by endophytic microbes, it is a prerequisite to have a molecular understanding of cell signaling occurring in plants during stress progression. Plants encounter several stressors during their lifecycle, and due to the sedentary nature, their escape is unavoidable. Consequently, during evolution, plants attain molecular and biochemical plasticity at different levels to develop adaptive strategies and cope with the stressful milieu. Despite tremendous differences in the physiology and morphology of plants belonging to different families, basic cell signaling during stress is largely conserved (Hotamisligil and Davis, 2016). Stress perception disturbs the cellular homeostasis that is driven by the cell signaling cascade, and eventually activates a reciprocate/adequate response.

### Physiological and molecular impact of drought stress

In the actual physio-biochemical sense, drought imparts a state of dehydration, which might not be the solitary effect of water scarcity rather it may also occur due to extreme temperature regimes, i.e., cold and heat stress. Although, a physical drought condition is the actual unavailability of water that is attributed to lower precipitation or irrigation, but the physiological drought does not comprise of water unavailability rather it is the inability of water uptake. Such incapability of plant cells to harness the freely available water is a result of one or the other physiological state of plants, exemplified by extreme cold, ionic level, excess fertilizer application or altered pH status, etc. (Vinocur and Altman, 2005; Passioura, 2007).

In response to drought stress exposure, plants either acquire a “resistance” or “escape” approach against it, depending on the eco-physiological aspect. The former strategy is further divided into the tolerance or avoidance mechanism to deal with the stressful event. To escape the drought, the plant accelerates its growth, reproduces, and develops seeds for the next propagation before the elevation of stress severity (Kooyers, 2015). Contrary to this, for drought avoidance, the plant reduces the transpiration rate to improve the water use efficiency (WUE). Such an increase in WUE for a particular duration of drought through the stomatal regulation is also accompanied by an increase in root to shoot ratio. While for the tolerant stratagem, plants accumulate diverse anti-stress proteins and osmolytes, modulate sugar metabolism as well as transport to stabilize the cellular integrity during water-deficit conditions (Salvi et al., 2018, 2022; Volaire, 2018; Ghosh et al., 2020).

The dehydration stress disturbs the homeostasis of carbon assimilation and energy transfer by electron excitation/utilization, which results in the accumulation of ROS. Under non-stressed conditions, the ROS are generated at the basal level, which is efficiently tackled/scavenged by the antioxidative machinery of the plants (Miller et al., 2010; Negi et al., 2017). Besides, ROS have also been ascribed a role to regulate adequate cell signaling during stress and programmed cell death (Petrov et al., 2015). As ROS are produced by the process of energy and electron transfer, it is likely to influence the redox status of cell and signaling cascades during different metabolic processes. The disturbed redox state of the cell leads to a rise in the ROS level beyond the threshold level that jeopardizes cellular functionality by oxidizing the macromolecules, such as lipids, DNA, RNA, and proteins thereby instigating extensive damage to these biomolecules (Dietz et al., 2016; Noctor and Foyer, 2016). To overcome the aforesaid damage, plants possess an efficient detoxifying system encompassing enzymatic and non-enzymatic antioxidative mechanisms. Additionally, plants also manifest leaf curling, epicuticular wax deposition, osmolyte accumulation to minimize the damage.

All these physio-biochemical alterations comprehend a dynamic yet highly regulated molecular response at a cellular level depending on the severity extent as well as the genotype being exposed to the stressor (Dinakar and Bartels, 2012). These molecular regulations are essentially governed by accumulation of different phytohormones and the interplay of their signaling cascades. The phytohormones like abscisic acid (ABA), jasmonic acid (JA), brassinosteroids (BR), cytokinin (CK) mostly play an unprecedented role in the modulation of the biological processes. Among diverse interacting layers of phytohormone response, ABA is extensively studied with an emphasis on drought stress response (Brossa et al., 2011). However, the recent research pertaining to phytohormonal regulation has unfolded the crucial role of other phytohormones and their molecular cross-talk in mediating drought stress tolerance response. Besides, drought stress also instigates different signaling components including MAPK and Ca^+2^ signaling, ROS, NO, SnRK2, etc. These molecular players trigger signal transduction and activate the expression of drought stress-responsive genes.

### Drought stress tolerance response mediated by endophytes

**FIGURE 1:**
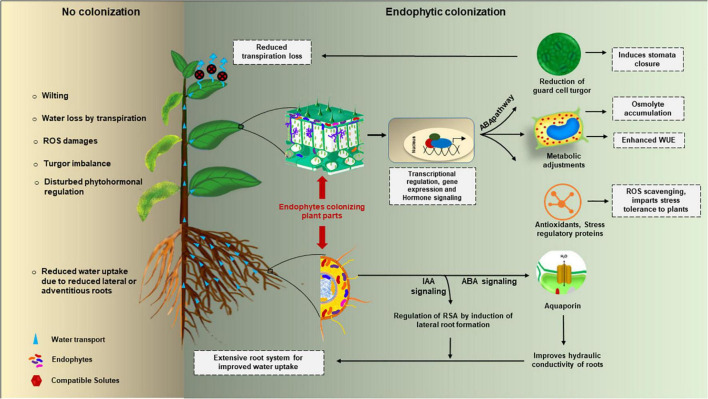
Endophytic mediated cell-signaling during drought stress response in plants.

Among abiotic stresses, drought stress deeply accounts for reduced plant growth and ultimately yield. To provide enough food to an ever increasing world population, it is extremely important to counteract the effect of drought stress on plants. The strategies which are presently being employed to reduce the effect of drought stress in plants are non-renewable and eco-destructive thus, to protect plants from drought stress, strategies engaging eco-friendly alternatives are the need of the hour. Studies reveal that endophytic microbes can be best used as an alternative to destructive fertilizers and pesticides to improve plant growth and yield. Myriad of endophytes live in a mutualistic relationship with plants and provide several benefits to plants. Some of them account for alleviating the effect of drought stress on plants. Different endophytes may employ different mechanisms to counteract the drought stress effect in plants (Figure 1) which are discussed below.

Since physiologically drought is the inability of the plant to uptake water, alterations in root physiology and morphology such as an increase in the number of roots, deeper root system and, small diameters of roots are some of the changes occurring in roots to increase the water uptake from the soil. Studies have found that endophytic bacteria (Table 1) produce several plant hormones such as indole acetic acid (IAA), ABA, and gibberellins, synthesize ammonia, increase the bioavailability of nutrients and shield the plants from pathogens to boost the increase in root length and density (Ullah et al., 2019). The reduced ability for water uptake may lead to a change in the osmotic potential of the cell. Once the disturbance in osmotic potential is sensed by the cell, it carries out osmotic adjustment by the accumulation of compatible solutes such as sugars (e.g., sucrose), glycine betaine, organic acids (e.g., malate), inorganic ions (e.g., calcium) and proline. Osmotic adjustment thus helps the plant to withstand damage induced by drought stress and to protect proteins, enzymes, cellular organelles, and genetic material from oxidative damage (Ullah et al., 2019). Many endophytic bacteria have been discovered (Table 1) that increase the level of compatible solutes inside the cell to protect the plant from drought stress. One such example is *Bacillus amyloliquefaciens*, an endophytic bacterium that has been found to increase the concentration of compatible solutes such as amino acids (phenylalanine, aspartic acid, glutamic acid, cysteine, and proline) to protect the rice host under stress conditions (Shahzad et al., 2017). Another parameter to check whether a plant can withstand drought stress-induced damage or not, is the relative water content (RWC) of plants. It is the estimation of actual water content in comparison to maximum water holding capacity and therefore, the larger the RWC values a plant exhibits greater is the ability of that plant to adjust under drought stress conditions. Several endophytes have been explored that increase the RWC in plants and help in mitigating the adverse effects of drought stress. Further, drought stress induces an increase in levels of ROS such as hydroxyl radical, hydrogen peroxide, superoxide anion radical, and singlet oxygen. These ROS are produced due to partial reduction of atmospheric oxygen and trigger oxidative damage to lipids, proteins, and macromolecules (Mittler, 2002). Several studies have revealed that endophytic bacteria and fungi (Tables 1, 2) can assist the host plant to decrease the levels of ROS by producing enzymes such as superoxide dismutase, peroxidase, and catalase which are involved in maintaining the basal levels of ROS. Moreover, the plant needs to maintain optimum growth to withstand the adverse effects of drought stress. Endophytic bacteria have a great role in producing plant growth-promoting hormones (Table 1) such as auxins, ABA, and ethylene which help the plants to tolerate drought stress. Auxins are an important group of phytohormones that are naturally produced by plants in the form of IAA and indole butyric acid. They are responsible for regulating different physiological processes in plants such as seed germination, cell division, cell elongation, cell differentiation, root development, photosynthesis, and shielding plants against stressful conditions. Studies reveal that auxins elevate tolerance in plants against drought stress (Ullah et al., 2019). Many endophytic bacteria have the potential to produce IAA and also intervene in the transportation of auxin inside the plant. With an increase in auxin concentration inside the plant, lateral root formation takes place which leads to a rise in the surface area of roots and thereupon more absorption of water and minerals from the soil. ABA is another important phytohormone that is responsible for regulating several morphological, physiological, biochemical, and molecular processes as well as growth and germination (Ullah et al., 2019). According to reports, ABA is also engaged in different signaling pathways that regulate stress-responsive genes (Egamberdieva et al., 2017; Salvi et al., 2020). Though ABA induces the development of roots, enhancement of their length and density to increase the contact of roots with more moisture deep inside the soil, there are only a few reports about endophytic microbes producing ABA and hence mediating drought tolerance through ABA production. Ethylene is a plant hormone that is involved in fruit ripening, senescence, and abscission. Drought stress leads to an increase in ethylene production inside the plant which ultimately impede the growth of plants. Many endophytic microbes have been identified that produce 1-aminocyclopropane-1-carboxylic (ACC) deaminase enzyme (Glick, 2014). ACC deaminase catalyzes the inactivation of ACC, the precursor of ethylene, and produces ammonia and α-ketobutyrate. Inactivation of ACC results in the decreased levels of ethylene inside the plants and ultimately increased plant growth. Editing in *Arabidopsis*-*Pseudomonas* holobiont targeting the alteration in ethylene synthesis *via* ACC synthase gene in gene and ACC deaminase in bacteria has revealed a promising model for plant nutrient enhancement to tackle increasing food demand (Ravanbakhsh et al., 2021). The activity of endophytic ACC deaminase may mediate increased plant growth under drought-stress conditions.

**TABLE 1:** Utilization of bacterial endophytes to improve stress tolerance response of host plants.

Sr. no.	Bacterial endophytes	Crops/plant	Mode of action/mechanism	Genes	References
1.	*Bacillus subtilis*	*Arabidopsis thaliana*	Increased production of proline	*proBA*	Chen et al., 2007
2.	*Arthrobacter sp.* EZB4 *and Bacillus sp.* EZB8	Pepper (*Capsicuum annuum* L.)	downregulation of the stress-inducible genes	*CaACCO* and *CaLTPI*	Sziderics et al., 2007
3.	*Bacillus licheniformis K11*	Pepper (*Capsicum annuum*)	Production of auxin and ACC deaminase by regulation of stress related genes	*Cadhn, VA, sHSP* and *CaPR-10*.	Lim and Kim, 2013
4.	*Azospirillum brasilense N040* and *Bacillus amyloliquefaciens 5113*	Wheat (*Triticum aestivum*)	Reduced levels of ascorbate peroxidase (APX1), S-adenosyl-methionine synthetase (SAMS1), and the heat shock protein (HSP17.8)	*APX1, SAMS1, HSP17.8*	Kasim et al., 2013
5.	*Pseudomonas aeruginosa* strain GGRJ21	Mung Bean (*Vigna radiata*)	Up regulation of drought stress responsive genes	*DREB2A, CAT1*, and *DHN*	Sarma and Saikia, 2014
6.	*Gluconacetobacter diazotrophicus*	*Saccharum officinarum* cv. SP70-1143	IAA and proline production	*ERD15 DREB1A/CBF3* and *DREB1B/CBF*	Vargas et al., 2014
7.	*Burkholderia phytofirmans PsJN*	*Solanum tuberosum* L.	Upregulation of cellular homeostasis, and the detoxification of reactive oxygen species	Extracytoplasmatic function (*ECF*) group IV sigma factors	Sheibani-Tezerji et al., 2015
8.	*Pseudomonas fluorescence RG11 Micrococcus*		Upregulation of melatonin and its intermediates (tryptamine, 5-hydroxytryptophan, serotonin, and N-acetylserotonin)	*VvTDC1, VvTDC2, VvTDC3, VvSNAT*	Jiao et al., 2016
9.	*Pseudomonas fluorescens, Enterobacter hormaechei*, and *Pseudomonas migulae*	Foxtail millet (*Setaria italica* L.)	up-regulation of 1-aminocyclopropane-1-carbox- ylate (ACC) deaminase gene (acdS) which cleaves the precursor of ethylene (ACC)	*acdS*	Niu et al., 2018
10.	*Bacillus Subtilis* and *Paenibacillus illinoinensis*	Pepper (*Capsicuum annuum* L.)	Increases the vacuolar osmotic pressure	*H^+^-PPase* (V-PPase)	Vigani et al., 2019
11.	*Streptomyces chartreusis* WZS021	Sugarcane	Modulation of root parameters, osmotic adjustment, phytohormone production	*proDH, Xanthine dehydrogenase*	Wang et al., 2019
12.	*Cellulosimicrobium sp. JZ28*	Desert plant (*Panicum turgidum*)	Osmotic adjustment	*nhaA, cspA, groEL, groES, dnaK, lexA, proABC*	Eida et al., 2020b

**TABLE 2:** Utilization of fungal endophytes to improve stress tolerance response of host plants.

Sr. no.	Fungal endophytes	Crops/plant/plant parts	Mode of action/mechanism OR physiological changes in plant	Genes	References
1.	*Glomus intraradices*	Soybean (*Glycine max* L. cv. Williams), lettuce (*Lactuca sativa* L. cv. Romana), maize (*Zea mays* L. cv. Prisma), and tobacco (*Nicotiana tabacum* L. cv. Samsun)	regulate both signaling pathways and also effector proteins involved in the final plant responses	*Gi14-3-3*	Porcel et al., 2006
2.	*Piriformospora indica*	*Arabidopsis thaliana*		*(RD)29A, (ERD)1*, *PLD*, (*DREB*)*2A*, (*SDIR*)*1*, (*CBL*)*1*, (*CIPK*)*3*	Sherameti et al., 2008
3.	*Piriformospora indica*	Chinese cabbage	Increase in CAS protein and upregulation of POX, CAT and SOD enzymes	*DREB2A, CBL1, ANAC072, RD29A*	Sun et al., 2010
4.	*Glomus intraradices*	Carrot roots	Upregulation of aquaporin genes	*GintAQPF1* and *GintAQPF2*	Li et al., 2013
5.	*Trichoderma harzianum* TH-56	*Oryza sativa*	Upregulation of aquaporin, dehydrin and malondialdehyde genes	DHN/AQU	Pandey et al., 2016
6.	*Piriformospora indica*	*Zea mays* L.	proline content increased, accumulation of malondialdehyde decreased, enhanced antioxidant enzyme activity	*DREB2A, CBL1, ANAC072, and RD29A* were upregulated	Xu et al., 2013
7.	*Piriformospora indica*	*Zea mays* L.	Plant hormone signal transduction	*TGA1, TGA9, AUX/IAA, MYB2, MYC2, DREB10NAC, AREB(bZIP)*	Zhang et al., 2018
8.	*Penicillium chrysogenum (62%), Penicillium brevicompactum (27%), Alternaria sp. (6%), Phaeosphaeria sp. (3%), and Eupenicillium osmophilum (2%)*	*Colobanthus quitensis*	Modulated the expression of genes related to ABA synthesis pathway	*CqNCED1, CqABCG25, CqRD22*	Hereme et al., 2020

## Omics based approaches for exploration of endophyte mediated drought tolerance

**FIGURE 2:**
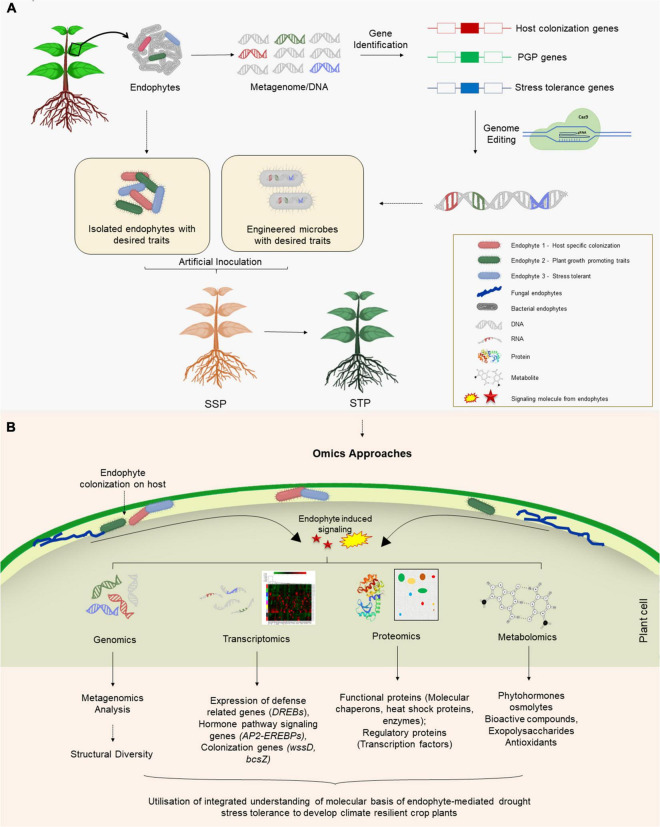
**(A)** Application of endophytes for mitigating drought stress in crop plants. Endophytes naturally inhabiting plants in water deficit regions may be isolated and genes involved in host colonization, growth promotion and stress tolerance can be identified. Through the genetic engineering/genome editing of the candidate gene(s), the information can be utilized to engineer microbes that have ability to colonize non-host crop plants, promote their growth and confer stress tolerance. Additionally, the naturally isolated endophytes can also be used to produce stress tolerant crop plants. **(B)** Application of omics-based approaches to understand molecular basis of endophyte-mediated drought tolerance.

Owing to numerous beneficial aspects of endophytes, it is crucial to understand the plant-endophytic interactions, especially the role and regulation of genes or proteins involved in the metabolic process and their evolutionary perspective. Such studies necessitate the information on the genome sequence of the host and endophyte as well, which will pave the way to engineer/manipulate the mutualistic relationship between the two. Numerous omics-based approaches including genomics, metagenomics, and functional genomics (Kaul et al., 2016) have dramatically revolutionized microbial studies and enabled the rapid and detailed assessment of the diversity, evolution, and molecular or biochemical composition of the microbial communities within a host plant (Figure 2). In the following sections, we discuss advancements in molecular perspectives of plant-endophyte relationships owing to the utilization of various omics-based approaches.

### Genomics

Whole Genome Sequences (WGS) of numerous endophytes are available in the public domain (FungiDB, NCBI) owing to the advancement in Next Generation Sequencing (NGS) methods. Whole-genome analysis (WGA) coupled with comparative genomics offers genome-scale identification of genes involved in host colonization, growth promotion, and protection against (a) biotic stresses among others (Table 3). WGA has facilitated the identification of several such genes in prevalent endophytes species belonging to *Bacillus* genus (Contreras-Pérez et al., 2019; Flores et al., 2020). Many whole genome sequence-based studies have identified genes implicated as monumental in drought stress tolerance (Table 3). Notably, many genes involved in successful interaction between endophyte and host plant may also be transposon-encoded. Transposon mutagenesis sequencing (TnSeq) is another technique employed for the identification of such genes (van Opijnen et al., 2009). TnSeq has revealed important information in several microbes including endophytes such as *Pseudomonas simiae* (Cole et al., 2017), *Azoarcus olearius*, and *Herbaspirillum seropedicae* (Do Amaral et al., 2020). In addition, quorum sensing molecules are essential during endophyte-plant or microbe-microbe interaction inhabiting the same host (Kusari et al., 2014). WGS of endophytes belonging to different genera has identified genes involved in the synthesis of such molecules (Parthasarathy et al., 2018). The utilization of quorum sensing molecules for enriching the microbiota of crop plants can be a useful approach toward sustainable agriculture. Further, the study of whole genome can be used to investigate the taxonomic classification and evolutionary aspects of plant-endophyte interactions. Pan-genome analysis also allows us to study a core genome (that is present in all strains across a species) along with an accessory genome (genes unique to the strain under consideration). This helps to identify the important cluster of genes which are accounted for the differences in growth, establishment, adaptation, and evolution of endophytic connotation. The absence of any evolutionary relationship of an unknown gene with known genes limits the genomics study as it does not contribute to the functional annotation.

**TABLE 3:** Omics-based studies on plant-endophyte relationship to study the endophyte mediated drought stress tolerance conferred to host plant.

S. No.	Host	Endophyte	Omics approach utilized	References
1.	Poplar	*Stenotrophomonas*	Genomics/WGS	Ulrich et al., 2021
2.	Desert plant (*Panicum turgidum*)	*Cellulosimicrobium*	Genomics/WGS	Eida et al., 2020b
3.	Pioneer desert halophytic plant (*Zygophyllum simplex*)	*Paenibacillus*	Genomics/WGS	Eida et al., 2020a
4.	*Colobanthus quitensis*	Endophyte consortia	Transcript profiling	Hereme et al., 2020
5.	Sugarcane	*Streptomyces chartreusis*	Genomics/WGS	Wang et al., 2019
6.	Poplar	Endophyte consortia	Genomics/WGS/metabolomics	Khan et al., 2016
7.	Barley	*Piriformospora indica*	Metabolomics/proteomics	Ghaffari et al., 2019
8.	Wheat	*A. sclerotigenum, S. implicatum*	Comparative metabolomics	Llorens et al., 2019
9.	Tall fescue	*Epichloë* species	Transcriptomics	Dinkins et al., 2019
10.	Soybean	*Sphingomonas*	Genomics/WGS	Asaf et al., 2018
11.	Maize	*Piriformospora indica*	Transcriptomics	Zhang et al., 2018
12.	Rice	*Trichoderma harzianum*	Transcript profiling	Pandey et al., 2016
13.	Potato	*Burkholderia phytofirmans*	Transcriptomics	Sheibani-Tezerji et al., 2015
14.	Sugarcane	*Gluconacetobacter diazotrophicus*	Transcriptomics	Vargas et al., 2014
15.	Barley	*Piriformospora indica*	Proteomics	Ghabooli et al., 2013
16.	*Arabidopsis thaliana*	*Pseudomonas chlororaphis*	Transcriptomics	Cho et al., 2013

This limitation can however, be resolved by metagenomics. Metagenomics is one of the important approaches to analyse the genomic constituent of the microbial community and the strategies adopted by them to encounter the surrounding factors. It assists in analysing the genome of organisms directly from the environment and reveals the possible function of genes or their participation in a particular biological pathway. NGS-based metagenomics studies coupled with *in silico* analysis provide direct information about exceptional enzymes and the function of unknown organisms. For example, metagenome sequencing has revealed important functions required for survival of bacterial endophytes inside plants (Sessitsch et al., 2012). Additionally, the colonization pattern of different tissues of the host can be traced through metagenomics. To obtain a more comprehensive view of the functions, mechanisms, and regulation of the microbiome under the stressful conditions we need to study the meta-transcriptomes, meta-proteomes or meta-metabolomes.

### Comparative genomics

Plant microbiome is usually composed of endophytic or non-endophytic strains. An omics-based comparison of endophytic and its non-endophytic counterpart will allow us to identify the crucial characteristics that are involved in the endophytic colonization (Lòpez-Fernàndez et al., 2015). Genomic features differentiating organisms with different lifestyles can be elucidated using comparative genomics (Mitter et al., 2013). For example, it aids in identifying regulatory genes involved in host penetration, colonization and establishment of symbiotic relationship. Such genes may be absent from genomes of non-endophytes. Lateral transfer of such genes from endophytic to non-endophytic may eventually take place in the form of mobile genetic elements such as plasmids, genomic islands or transposons (Taghavi et al., 2010; Tisserant et al., 2013). Moreover, comparative genomics may also help in discriminating between pathogenic and non-pathogenic strain. Since both these organisms possess the capability of invading the plant host, comparative genomics identifies genes responsible for pathogenicity or the lack of it (Yang et al., 2019). Hence, several differences have been highlighted between the pathogenic and non-pathogenic lifestyles of endophytes with the help of studies utilizing comparative genomics. For example, non-pathogenic strains have genes enriched in biosynthetic processes while pathogens have predominance of genes involved in degradation (Karpinets et al., 2014). Comparative genomics may also shed light on the molecular basis of host range for a given endophyte. Bacteria with large genomes usually colonize a wide range of host often unrelated to each other (Mitter et al., 2013). Moreover, comparison of genomes of endophytes isolated from hosts belonging to different ecological niches may reveal important features providing adaptive advantages to these endophytic organisms (Yi et al., 2017). Altogether, comparative genomics has revealed genes related to colonization of host plants (such as those involved in motility, chemotaxis), establishment of symbiotic relationship (signal transduction and transcription regulation) and conferring stress tolerance to host (enzymes, hormones) or those involved in pathogenesis (secretion systems). Such information can be methodologically applied for designing microbes endowed with colonization abilities that can promote plant growth and provide drought stress tolerance to the host (Figure 2A).

### Functional genomics

To examine interactions between an endophyte and the plant, it is important to understand how two genomes interact with each other. For that, it is important to investigate the expression of genes from two genomes simultaneously which is possible using the dual RNA-seq technique. Moreover, the interaction among the endophytes present within the same host can also be explored by comparative transcriptome analysis. Expression studies, under different stress conditions, can unveil putative candidate genes responsible for stress tolerance/sensitivity whose function can be targeted for improved tolerance in the future (Figure 2). Gene expression profiling can be achieved through transcriptomic analysis using RNA-seq, microarray, SSH, or SOLiD-SAGE techniques however, each has its own set of advantages (Wang et al., 2019).

Mass spectrometry-based proteomics and/or metabolomics studies also provide an efficient platform for post-genomic analyses. Plant-endophyte interactions result in the production of different proteins and metabolites as compared to the non-infected plant. Proteomic and metabolic profiling can help in investigating the new pathways involved in the production of these proteins and metabolites in infected plants with endophytes. These novel or bioactive metabolites could help the plant in mitigating stress (Yan et al., 2019). Moreover, the changes in metabolomes often regulate the switch in the lifestyle of the microbes for example switch from epiphytic to endophytic or vice versa. Interestingly, based on functional genomics revelations (Table 3), endophytic fungi have been found to be different from endophytic bacteria in their mode of functioning under drought stress. The fungi such as *P*. *indica* enhance the levels of phytohormones (auxins, ABA, SA and cytokinins) and regulate the expression of stress responsive genes in maize (Zhang et al., 2018) while endophytic bacteria (*Gluconacetobacter diazotrophicus)* suppress the accumulation of these hormones in sugarcane roots and activated ABA-dependent stress signaling in shoots (Vargas et al., 2014). Similarly, *Pseudomonas chlororaphis*, colonized *Arabidopsis thaliana* plants also showed downregulation of ABA and ethylene signaling (Cho et al., 2013). However, *T. harzianum* in rice and *Burkholderia phytofirmans* in potato plants increased the expression of genes involved in redox homeostasis (Sheibani-Tezerji et al., 2015; Pandey et al., 2016).

## Conclusion and future outlook

Drought is one of the major stressors, which affect global crop productivity. The impact of water scarcity has severely implicated diverse aspects of crop yield. Hence there is an urgent need to deal with the issue of food insecurity/safety by improving crop productivity and producing high-quality food under stressful conditions. Besides, climate change and anthropogenic activity further raise several concerns for adequate crop production. This necessitates the climate-resilient crops which possess a higher potential to withstand extremes of conditions. The improved productivity needs to be balanced within the diminishing agricultural land and water resources. In nature, it is often observed that some plants exhibit a broad suite of stress resistance compared to others. Although numerous approaches have been adopted by researchers to understand the underlying mechanism of differential behavior of susceptibility and tolerance, but the role played by the plant microbiome has attained very less attention in this regard.

Plant microbiomes may contain different sorts of organisms including bacteria and fungi that display an intimate association with plants and might play a significant role in host stress tolerance. Therefore, it is important to understand the crucial role of endophytes in stress response and utilize them as an efficient tool to enhance the tolerance potential of the host plant. For this, it is important to identify the signature gene/protein that could have beneficial implications for the stress response. There is a critical debate about how plants mediate the plant-endophyte interaction in parallel to confronting the pathogenesis by restricting the pathogen attack. What are the specificities of different receptors, that account for the differential recognition of microbes? During evolution, plant microbes have developed an intricate association of mutualistic or antagonist nature depending on their survival benefits. It is important to broaden our knowledge about the evolutionary aspects of both plants and endophytes which aids in their beneficial interaction.

The continuous advancement in the tools and techniques of functional and genomics studies has uncovered several aspects of plant-microbe interactions. Furthermore, techniques such as fluxomics which connects the genomic and metabolic activities and integrates the cellular functional output with the plant phenotype (Salon et al., 2017) have not been efficiently applied to plant-endophyte interactions. A combined fluxomics and transcriptomics study revealed increased expression of genes involved in general stress response in *Gluconobacter oxydans* during different growth phases (Hanke et al., 2013). Notably, *Gluconobacter* is one of the most common endophytic genera, therefore, similar studies on endophytes and their hosts under stress can reveal the flux through different metabolic pathways highlighting the real picture of endophyte mediated stress tolerance at cellular/metabolic level. The sequence information of genes further helps to understand possible functions and to disclose its implication toward a particular trait of endophyte for the host plant. Additionally, the availability of several open-source bioinformatics tools and software has further embarked the big data analysis. Overall, there are enormous unexplored aspects of endophytism that would be instrumental in developing an intrinsic stress tolerance response and developing climate-resilient crop plants in the future. In addition, endophytism could also aid in curtailing the usage of harmful chemical fertilizers thus encouraging eco-friendly farming and sustainable agriculture.

## Author contributions

PS conceptualized and wrote the first draft of manuscript. Kajal, HM, RA, and PS involved in data curation, tables or figures preparation. RD, VG, and PS edited the final manuscript. All authors contributed to the article and approved the submitted version.
